# Beyond Problem-Solving: Interactive Worked Examples as Pathways to Dual Teacher Expertise

**DOI:** 10.3390/jintelligence14090203

**Published:** 2026-09-01

**Authors:** Tikva Ovadiya

**Affiliations:** Department of Mathematics Education, Oranim Academic College of Education, Kiryat Tivon 3600600, Israel; tikva_o@oranim.ac.il

**Keywords:** cognitive load theory, interactive worked examples, non-routine mathematics problems, problem-solving, pedagogical knowledge, in-service mathematics teachers

## Abstract

Teacher learning presents a unique cognitive challenge: educators must simultaneously develop mathematical expertise and pedagogical knowledge for teaching. This study examines how interactive worked examples function as cognitive tools to support this dual learning process. Using a qualitative, exploratory case-study design, I investigated how 30 in-service mathematics teachers engaged with interactive worked examples of non-routine problems in a professional development setting designed to reduce extraneous cognitive load while promoting germane processing. Drawing on Cognitive Load Theory, expertise development frameworks, and recent perspectives on cognitive offloading and affective engagement in multimedia learning, I analyzed the cognitive and emotional mechanisms underlying teachers’ acquisition of both mathematical content knowledge and pedagogical content knowledge. Results from teachers’ journals, reflective protocols, and self-assessed cognitive load measurements reveal three key findings: First, teachers experienced analyzing worked examples not as passive observation but as active problem-solving, engaging in self-explanation and schema construction. Second, the interactive format enabled them to discover new mathematical relationships and solution strategies that were previously unavailable to them. Third, and notably, teachers simultaneously developed pedagogical insights—recognizing instructional approaches and anticipating student difficulties—while working through the mathematical content. These findings extend worked example theory by demonstrating that carefully designed interactive examples can support dual-domain learning in professional contexts. By managing cognitive load through scaffolded interactivity, worked examples enable teachers to construct both mathematical and pedagogical schemas concurrently, suggesting a cognitively efficient pathway for teacher professional development. Given the study’s exploratory, single-setting design, these mechanisms are offered as a theoretically grounded account to be tested in future comparative research rather than as definitive causal claims.

## 1. Introduction

In-service mathematics teachers (ISMTs) are expected to cultivate a range of competencies in their students, including the ability to solve non-routine mathematical problems—problems that deviate from familiar procedures and demand flexible thinking and creative strategy use (Borromeo Ferri, 2018; Cevikbas et al., 2022; Schukajlow et al., 2021). Supporting students in developing these competencies poses considerable instructional challenges, particularly when teachers themselves lack opportunities to engage meaningfully with such problems.

Research underscores the value of involving ISMTs in authentic non-routine problem-solving experiences as a means of strengthening their mathematical understanding, pedagogical content knowledge (PCK), and adaptive expertise (Borromeo Ferri, 2018; Cevikbas et al., 2022; Schukajlow et al., 2021). Nevertheless, many teachers report uncertainty regarding appropriate instructional strategies, assessment techniques, and how to scaffold students’ engagement in this domain (Nguyen et al., 2020). This gap was also evident in a pilot study conducted by the author with 25 ISMTs, which revealed confusion and lack of confidence in teaching non-routine problems, further emphasizing the need for well-structured professional development opportunities.

Although prior research has established the effectiveness of worked examples for novice learners in classroom environments (Sweller & Cooper, 1985; Chawla et al., 2021), the unique challenges encountered by practising teachers—who must both construct new mathematical schemas and develop pedagogical content knowledge for teaching non-routine problems—have been comparatively underexplored in the professional development literature. Most existing studies focus on worked examples as tools to enhance student learning, with limited attention given to their role in fostering teacher professional growth or addressing the dual cognitive demands that arise when teachers are required to learn new content and concurrently consider instructional strategies. This study seeks to fill that gap by examining how interactively designed worked examples, grounded in cognitive load theory and multimedia learning principles, serve as cognitive tools to support this dual learning process in a graduate-level professional development context.

The present study addresses this need by designing and implementing a graduate-level course in mathematics education that integrates interactive worked examples (IWEs)—digital, step-by-step solution sequences that actively engage learners. These examples draw on principles from cognitive load theory (Sweller, 2010), multimedia learning (Mayer, 2014), and mathematics education research on the use of worked examples in teacher learning environments (e.g., Barbieri et al., 2023; Ovadiya, 2018, 2022).

IWEs have demonstrated efficacy across educational settings, particularly in mathematics, where they enhance learners’ conceptual understanding and problem-solving skills. A meta-analysis conducted by Barbieri et al. (2023) provides further evidence that worked examples—used alone or paired with practice problems—are highly valuable for promoting mathematics learning across a wide range of topics.

This study contributes to the literature in two main directions:

- It examines how ISMTs experience and learn within an IWE-based instructional environment, focusing on their engagement with non-routine mathematical problems and the pedagogical insights they extract from the process.
- It applies IWEs within a theoretically grounded instructional design framework tailored to the professional learning needs of ISMTs, combining elements of cognitive science, multimedia theory, and mathematics-specific pedagogical approaches.

By examining ISMTs’ engagement with IWEs during non-routine problem solving, this study provides insights into how teachers develop both mathematical and pedagogical skills. The results are intended to guide the design of future professional development programs that prepare teachers to confidently and effectively teach non-routine mathematical tasks.

## 2. Theoretical Background

This section focuses on cognitive load theory and its connection to human learning, worked examples for problem-solving, learning with interactive worked examples, and element interactivity. It further addresses the nature of non-routine mathematical problems and the conceptual framework informing this study.

### 2.1. Cognitive Load Theory

Cognitive load refers to the effort required in working memory, which is the amount of information our working memory can process at any given time. One effective way to enhance learning while enabling more focus on peripheral and didactic issues, as opposed to solely solving the problem at hand, is by reducing cognitive load (CL). According to research in the fields of instructional design and pedagogy, there are three types of cognitive load: intrinsic cognitive load, which pertains to the effort required for a particular subject matter; extraneous cognitive load, which concerns how data or tasks are presented to a learner; and germane cognitive load, which relates to the cognitive effort needed to make sense of new information and integrate it with existing knowledge. Over the years, the additivity of these types of CL has been investigated and questioned. It is believed that they circularly influence each other (Orru & Longo, 2019). Some theories claim that if the learning environment is designed to reduce external CL as much as possible, the internal load can be more focused (Sweller, 2011), and the learner will devise a more effective schema about the subject. Cognitive schemata are mental structures that allow individuals to organize and retrieve complex information efficiently, reducing working memory load during problem solving (Sweller, 2010). In mathematics education, developing schemata is crucial for solving non-routine problems, as it allows learners—and teachers—to recognize underlying structures and apply suitable strategies flexibly (Borromeo Ferri, 2018).

Interestingly, CL theories are not generally considered in graduate teacher-training programs. This may be due to the assumption that such learners have already developed attention and learning habits appropriate for specific learning situations. However, this cannot be assumed, given the heterogeneity of learners in academia. Therefore, I aimed to reduce the ISMTs’ CL by making it a goal in the course design. I planned to achieve this by using specially designed applets (with GeoGebra (International GeoGebra Institute, Linz, Austria), Desmos (Desmos Studio PBC, San Francisco, CA, USA), or other interactive applications) to help learners analyze interactive WEs of problems and understand the key ideas underlying each solution. The rationale was that if learners expend less cognitive effort on solving the problem itself, they can invest more effort subsequently in discussing the didactic and pedagogical aspects of the problems in plenary sessions.

CL theory posits that optimal learning occurs when an individual can organize their thoughts through connections based on a schema that encompasses all aspects of knowledge within it (Sweller, 2011). This principle builds on the well-known theory of “the magical number seven, plus or minus two” (the number of items an average human can hold in working memory is 7 ± 2) (Miller, 1956), and Cowan’s assertion that the focus of attention holds only about four items (Cowan, 2001). Sweller further argued that CL is affected by the available resources of memory when a person performs a task. Therefore, for optimal learning, the learning environment must be designed to match the characteristics of human consciousness and memory (Sweller, 2016).

Phan et al. (2017) propose a framework that integrates cognitive load theory with the concept of achieving learners’ “optimal best” in mathematical problem solving. They emphasize the instructional efficiency of using worked examples, particularly for novice learners, as a means to reduce extraneous cognitive load and support schema construction. Their findings highlight the importance of managing element interactivity and aligning instructional design with learners’ prior knowledge to avoid the expertise reversal effect. The study also underscores the motivational dimension, showing that lower cognitive load can enhance learners’ belief in their capacity to reach higher levels of understanding and performance. Although this study centers on in-service mathematics teachers, the design of the learning environment must still account for their cognitive processing capacities. Phan et al. (2017) emphasize that cognitive load theory is broadly applicable across learner populations, including experienced individuals engaging with unfamiliar or complex tasks. In such contexts, teachers may adopt the cognitive stance of novices, especially when encountering non-routine mathematical problems or novel pedagogical frameworks. Accordingly, the use of instructional strategies—such as interactive worked examples—that manage intrinsic load and reduce extraneous load can facilitate meaningful schema construction and promote instructional efficiency. The integration of these design principles into professional development programs can support teachers in achieving “optimal best,” enabling both content mastery and pedagogical advancement.

A complementary perspective on cognitive load management comes from recent work integrating embodied cognition with CLT. Zou et al. (2025) propose that cognitive offloading—the use of external tools and representations to reduce working memory demands—can play a productive role in learning when it enables learners to redirect cognitive resources from lower-level procedural operations toward deeper schema construction. This framework is particularly relevant to the present study: the interactive applets functioned as cognitive offloading devices, externalizing dynamic mathematical representations and thereby allowing teachers to attend to the structural and pedagogical dimensions of non-routine problems rather than managing all computational elements simultaneously. Importantly, Zou et al. (2025) also challenge a strictly linear view of CLT, noting that brief periods of elevated cognitive load within a learning session are not necessarily detrimental and may, under certain conditions, support deeper understanding. This insight extends earlier frameworks by acknowledging the context-dependent and fluctuating nature of cognitive load in real-time learning environments, a dynamic that is clearly evident when experienced teachers encounter genuinely unfamiliar mathematical content.

### 2.2. Problem-Solving Using Worked Examples

The present study began with the premise that ISMTs need clues to arrive at the complete solution and then discuss suggestions for practices to teach the problem. The clues in the current study came via a WE that appeared in an app.

Originating from a classic study by Sweller and Cooper (1985), the WEs effect suggests that having learners study worked-out problem solutions is more effective for learning than problem-solving practice alone in terms of reducing problem-solving errors and increasing performance on mathematics assessments. This seminal work served as a springboard for thousands of new studies on the WEs effect (Booth et al., 2015; Renkl, 2014) with examinations of the effects of a wide range of variations (e.g., Sweller, 2012).

Studies have shown that analyzing WEs boosts a learner’s ability to solve problems more than just learning problem-solving strategies. Evidence indicates that learning through WEs enables generalization or abstraction of the concept being studied and helps transfer understanding and strategies from one problem to another, even across different mathematical topics (Hoogerheide et al., 2016; Sweller, 2015). Examining and interpreting WEs reduces cognitive load (CL) and extends the capacity of working memory in real time. In fact, WEs effectively lower CL and may encourage the development of appropriate cognitive schemas to help solve similar problems later (Kirschner et al., 2006). Chawla et al. (2021) conducted a thorough meta-analysis showing that worked examples significantly improve mathematical problem-solving across various learner groups and domains. Their results indicate a moderate overall effect size, with particular benefits for learners engaging with faded worked examples, which gradually reduce instructional guidance to promote independent problem solving and long-term transfer. Notably, the study emphasizes the added value of interactive digital worked examples, noting that features like feedback, manipulation, and staged progression enhance learners’ engagement, cognitive processing, and self-regulation. These insights are very relevant to the current research, which investigates how in-service mathematics teachers interact with interactive worked examples of non-routine problems. Although these teachers are experienced practitioners, they may act as novices when confronting unfamiliar mathematical content or pedagogical methods. Therefore, the cognitive and motivational benefits identified by Chawla et al. (2021) support the use of interactive, well-designed worked examples as an effective way to help teachers learn in a multimedia professional development setting.

### 2.3. Learning from Interactive Worked Examples

A static WE is shown on a page or screen and is accompanied by a verbal or written description of the solution stages. It may or may not include accompanying illustrations, graphs, or additional explanations. An interactive WE, on the other hand, uses dynamic software to demonstrate and manage the mathematical phenomena and solutions.

Atkinson and Renkl (2007) identified three interactive environments that encourage analysis of WEs: incomplete examples, examples requiring self-explanation, and dynamic examples. Incomplete examples require the learner to fill in information; the amount of critical information provided is reduced as the learner’s knowledge develops. Examples requiring self-explanation present the mathematical solution, and the learner is prompted to add appropriate mathematical proofs at all stages of the solution. It demands an in-depth understanding of the algorithm or the particular stage in the solution process. Dynamic examples present the entire solution and are unaccompanied by explanations. Such processes effectively enhance learners’ perceptions and their ability to transfer learning to other problems (Hoogerheide et al., 2016). In addition, it has been shown that learners using interactive WEs suffer fewer distractions and achieve more significant learning (Tindall-Ford et al., 2015), and dynamic visual representations encourage the learner to pay attention to critical features, thus reducing CL during learning and freeing up memory to understand the mathematical complexity in terms of prior knowledge and to create new knowledge (Yung & Paas, 2015).

The affective dimension of engagement with interactive learning materials is increasingly recognized as integral to, rather than separate from, cognitive processing. Weber et al. (2023) examined how different types of reflection stimuli influence pre-service teachers’ emotions, immersion, cognitive load, and knowledge-based reasoning. Their findings demonstrate that positive emotions—particularly enjoyment and engagement—positively predict germane cognitive load and immersion in learning tasks, while negative emotions such as boredom and anxiety suppress both. This relationship carries direct implications for IWE-based professional development: when learners encounter challenging interactive content that initially generates frustration or uncertainty, the subsequent emotional shift toward engagement and a sense of accomplishment can activate the very germane processes that drive schema construction. Weber et al.’s (2023) framework thus suggests that the emotional trajectory through a learning episode matters as much as the cognitive load level itself. For teacher learners engaging with complex non-routine problems through dynamic applets, emotional engagement and immersion may serve as critical mediators between high intrinsic load and productive learning outcomes.

### 2.4. Element Interactivity, Complexity, and Interactive Worked Examples

Element interactivity measures the complexity of a concept and is a fundamental part of cognitive load theory, usually applied to biologically secondary information (Chen et al., 2023). An element is defined as a concept or procedure that needs to be learned but can be broken down further based on the learner’s level of expertise. “Interactivity” refers to the degree of inherent connection between elements that require simultaneous processing in working memory to understand. (In contrast, non-interacting elements can be processed separately without referencing each other). The more elements that must be handled at the same time by working memory, the higher the element interactivity. However, element interactivity does not give an exact measure of complexity because an individual’s experienced complexity depends on both the nature of the information and their long-term memory content. Most effects of cognitive load theory disappear or even reverse when students are processing information with low element interactivity, leading to the expertise reversal effect (Chen et al., 2017; Chen et al., 2023); as element interactivity drops, the instructional effects diminish and may even turn negative. Therefore, recommended teaching methods largely depend on the element interactivity of the learning task.

Importantly, the suitability of an instructional procedure does not only depend on the nature of the content, but also on the learner’s prior knowledge. Thus, considering the element interactivity of a problem—or any measure of complexity that reflects the structure of human cognitive architecture—is essential to designing viable instructional environments. In this regard, Barbieri et al. (2023) provide compelling empirical support for the use of interactive digital worked examples in mathematics education. Their meta-analysis demonstrates that worked examples delivered via computer-based or interactive platforms are generally more effective than static, paper-based ones. Although interactivity was not the sole focus of their analysis, the researchers emphasize that digital formats with embedded feedback, navigational control, and faded guidance can enhance cognitive engagement and learning outcomes, particularly when aligned with cognitive load theory and multimedia learning principles. These interactive features serve to regulate cognitive processing, promote the sequencing of complex steps, and support self-regulation during problem solving.

Therefore, the current study applies these theoretical and empirical recommendations by qualitatively exploring the learning experiences of in-service mathematics teachers (ISMTs) as they work with interactive worked examples (WEs) based on non-routine mathematical problems.

### 2.5. Non-Routine Mathematical Problems

Routine mathematical problems are typically structured to provide students with practice in applying specific procedures demonstrated during instruction (Schoenfeld, 2016). These problems generally follow a predictable progression: (i) the task introduces a technique, (ii) the teacher models the technique, and (iii) students are given additional problems to practice and consolidate the skill. In contrast, non-routine problems require learners to devise novel methods or apply familiar knowledge in unfamiliar contexts. From this perspective, the classification of a task as routine or non-routine often depends on the learner’s prior experience. Boesen et al. (2010) found that students confronted with routine tasks tend to rely on imitative reasoning, whereas non-routine tasks prompt a broader array of reasoning strategies and deeper cognitive engagement.

Other scholars have proposed related frameworks. Pehkonen et al. (2013) distinguish between standard tasks, where the appropriate procedures are immediately apparent, and non-standard tasks, which are rarely encountered in conventional textbooks and may lack clearly defined solution paths. Krems (2014) further argues that solving non-routine problems requires training cognitive flexibility. For an information-processing system to adapt to dynamic problem environments, it must not only be capable of rapidly acquiring new skills, but also able to transfer prior knowledge and adjust strategies accordingly. Effective problem solving in such contexts often involves transforming one’s perspective, reconfiguring mental representations, or developing entirely new approaches.

Recent research has examined strategy use and flexibility as central components of non-routine problem solving. Elia et al. (2009) distinguish between inter-task flexibility—the ability to apply different strategies across different problems—and intra-task flexibility—the capacity to shift strategies within the same problem. According to Arslan and Yazgan (2015), strategy flexibility encompasses both the selection of the most appropriate method and the ability to switch strategies dynamically during the problem-solving process. Supporting this view, Gavaz and Yazgan (2021) demonstrated that instruction explicitly focused on non-routine problem solving can enhance students’ strategy flexibility and overall achievement. However, their study also revealed persistent challenges rooted in traditional mathematics education, which often emphasizes routine procedures over exploratory or flexible approaches. They recommend incorporating non-routine tasks more consistently in school curricula and teacher training to help students develop strategic adaptability.

The present study aligns with these recommendations by creating a professional learning environment in which in-service mathematics teachers engage directly with non-routine problems and reflect collaboratively on the problem-solving process.

### 2.6. Conceptual Framework

The present study draws on three complementary theoretical perspectives that informed the design of the professional development environment for in-service mathematics teachers: Cognitive Load Theory (Sweller, 2015), Cognitive Theory of Multimedia Learning (Mayer, 2014, 2021), and a Task-Based Approach to Teacher Learning (Zaslavsky, 2008).

Cognitive Load Theory (CLT) posits that instruction must align with the architectural structure of the human cognitive system, particularly working memory limitations (Sweller, 2015). Instructional materials and learning environments should therefore be designed to reduce extraneous load, manage intrinsic load, and promote germane processing. This study applies CLT to reduce unnecessary cognitive effort when teachers engage with complex non-routine mathematical problems, thereby supporting more efficient and effective learning.

Building on CLT, Mayer’s Cognitive Theory of Multimedia Learning identifies three core cognitive processes that underpin meaningful learning: selection, organization, and integration (Mayer, 2021). Learners first attend to relevant verbal and visual information (selection), then mentally organize that information into coherent representations (organization), and finally integrate verbal and visual modalities to construct a unified understanding (integration). Multimedia design that presents narration and synchronized visual animations can enhance these processes by aligning instructional stimuli with how the mind processes multimodal input (Mayer, 2014). Three principles guide this design: (1) “Less is more”—reduce extraneous processing by eliminating redundant or irrelevant information; (2) “More is more”—encourage deep, generative processing to foster connections and personal engagement; and (3) “Focused more is more”—foster generative processing while controlling extraneous load through directed attention and clear guidance.

Zaslavsky’s (2008) framework for task design in teacher education emphasizes the potential of well-crafted mathematical tasks to support teachers’ professional growth. She outlines three criteria for task design that promote meaningful engagement: (1) Adaptability—encouraging teachers to modify tasks, explore new instructional strategies, and demonstrate flexibility in planning; (2) Awareness of similarities and differences—developing teachers’ capacity to compare and classify mathematical objects and to explore the relationships among them; and (3) Coping with dilemmas and conflicts—equipping teachers to address pedagogical challenges and enhance students’ problem-solving abilities. Additionally, Zaslavsky stresses the importance of integrating technology, real-world contexts, and professional resources into instructional tasks. Teachers must also be able to identify and help students overcome cognitive barriers, such as difficulty interpreting representations, limited prior knowledge, or personal challenges.

**Figure 1:**
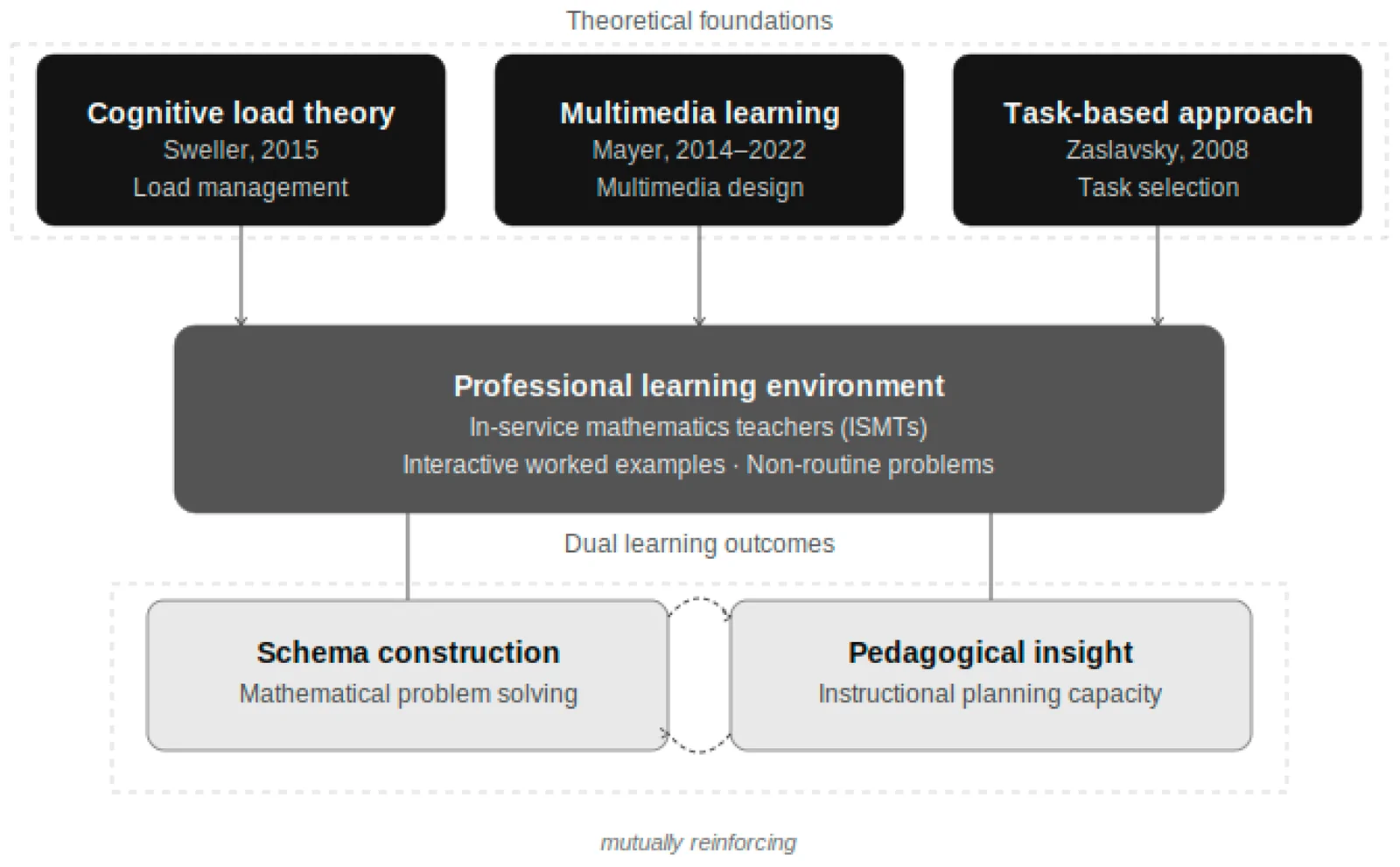
Conceptual framework integrating Cognitive Load Theory (Sweller, 2015), the Cognitive Theory of Multimedia Learning (Mayer, 2014, 2021), and task design for teacher learning (Zaslavsky, 2008).

To summarize, this study integrates Sweller’s, Mayer’s, and Zaslavsky’s theoretical models to construct a multimedia-based, cognitively informed, and task-driven learning environment. The study examines how this environment shapes in-service teachers’ mathematical and pedagogical development, particularly their capacity to solve non-routine mathematical problems in ways that reduce cognitive load while fostering deep engagement and adaptive instructional thinking. Figure 1 presents a conceptual diagram mapping these three theoretical frameworks onto the key components of the learning environment and the anticipated dual outcomes (mathematical schema construction and pedagogical insight development). In brief, CLT principles inform the IWE design by targeting load reduction; multimedia learning principles govern the representational format and interactivity of the applets; and Zaslavsky’s task-design criteria shape the selection, sequencing, and reflective follow-up of the problems. Together, these create the conditions for both mathematical and pedagogical learning to occur concurrently.

## 3. Method

### 3.1. Research Questions

In a preliminary pilot study conducted with 25 in-service mathematics teachers, participants were asked whether and how they address non-routine problems in their teaching. The results consistently revealed that teachers felt underprepared in this area and expressed a clear expectation for a structured professional development program to build their skills and practices for teaching non-routine mathematical problems.

The current research questions are organized around three theoretical frameworks, as shown in Table 1.

### 3.2. Research Goal

This study aims to explore how in-service mathematics teachers experience and learn in a multimedia-supported professional development environment that features interactive worked examples of non-routine mathematical problems. It seeks to understand how such an environment can enhance teachers’ mathematical content knowledge, pedagogical reasoning, and cognitive processing by drawing on principles from cognitive load theory, multimedia learning theory, and task design for professional growth.

### 3.3. Participants

The advanced graduate course was offered in each of three semesters, and in each, ten mathematics ISMTs (high school) participated in the study, yielding a total of 30 participants. Note that the 25 teachers mentioned in Section 3.1 refer to an earlier, separate pilot study; the two cohorts do not overlap. Participants were recruited through purposive sampling via the graduate programme’s regular enrolment process, as the study was embedded in an existing professional development context. The group comprised women and men, with teaching experience ranging from 2 to 20 years. All hold at least a bachelor’s degree in mathematics or a related discipline. Prior familiarity with GeoGebra or Desmos varied: approximately half reported occasional prior use, while the remainder had little to no prior experience with these tools. The participating ISMTs were heterogeneous in terms of culture, nationality, religion, age, and grade level taught, so even though they all taught in the same country, they brought notable diversity to the study.

### 3.4. Process

The ISMTs were assigned one, two, or three problems to solve in each lesson, accompanied by interactive WE. The non-routine problems were unfamiliar to the ISMTs, and sometimes the external structure was unique. The graduate course ran across one academic semester (approximately 14 weeks) per cohort, with weekly sessions of three academic hours each, yielding 13 instructional sessions per semester. In each session, the instructor—who was also the researcher—introduced the IWE context briefly, after which participants worked independently or in pairs on one to three problems. After the ISMTs solved each problem with the help of the interactive applet, they completed a questionnaire to assess and reflect on their CL. Then, they participated in semi-structured classroom discussions about their solutions, their CL experience, the reasons for their CL score, and any other ideas they might have for practices that could be used to teach the problem.

### 3.5. Applet Design—The Interactive Worked Examples

The applets were generally executed using GeoGebra (Classic 6) or the Desmos online graphing calculator. They allowed the learner to observe the problem in various situations that were pre-programmed by the applet’s designer. Some of the applets presented complete solutions; others presented the principal idea. Across all 26 IWEs, a consistent set of design principles was applied: (1) dynamic representation—each applet presented at least one manipulable element (e.g., a slider for a parameter, a draggable point) so that learners could observe how mathematical objects change continuously; (2) partial disclosure—applets revealed the key idea or visual structure of the solution without providing a complete formal proof, thereby preserving productive challenge; (3) multi-representational linking—wherever possible, applets displayed at least two coordinated representations (e.g., algebraic expression and graph) that updated simultaneously; and (4) minimalist interface—extraneous visual elements were suppressed to reduce split-attention effects. The specific problem whose data are reported in detail in this paper was selected because it exemplified all four design principles and generated the widest variety of documented learning responses across participants, thus affording the richest basis for illustrating the learning environment’s mechanisms.

#### Example Problem and Applet

Over the semester, the participants had to solve 26 problems. This paper focuses on the specific problem below to demonstrate the attributes of the learning environment as reflected by the data analysis. A single problem was selected for the demonstration to simplify understanding for the reader. This problem offered various aspects to help comprehend the learning experience of the course participants.

Show the solution of the following equation graphically:

*x*^2^ + *y*^2^ + 4*x*·*cos α* + 8*y*·*sin α* + 10 = 0 (1)

Given: 0 ≤ α < π.

Supportive information: Circle applet (GeoGebra): https://undergroundmathematics.org/trigonometry-triangles-to-functions/r7378 (accessed on 5 June 2019)

**Figure 2:**
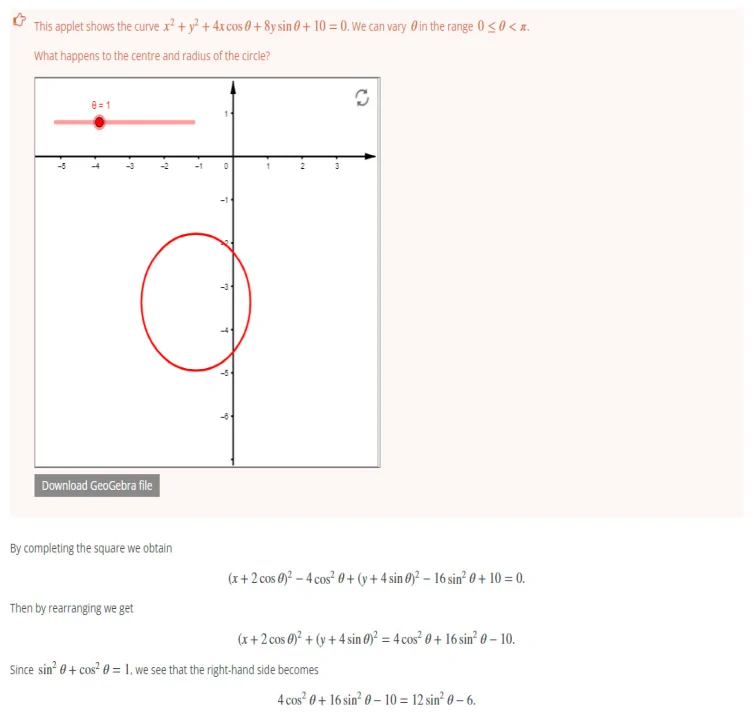
Screenshot of the circle applet provided for the ISMTs describing different circles based on the value of angle α. It allowed them to experiment with the equation, but did not provide the solution.

The task: Writing proof or solving the equation or both. See Figure 2.

The app’s unique feature was that it only displayed a visual representation of the solution to the problem, but not a complete solution, as is standard with WEs. Instead, the learner had to explore the dynamic graph of the circle, understand what they were observing, and then write a formal solution on their own.

With respect to Mayer’s design principles (see Section 2.6), this app is “less is more” because you can see only one circle; it can also be used as “more is more” because ISMTs should be curious to understand both the essence of the rule and the reason for the disappearance of the circle; and it is also “focused more is more” due to the challenge it offers to understand in depth the relationship between the circle and the sine function and the relationship between the graphic representations of both.

This problem focuses on solving an equation that describes a circle whose center and radius depend on angle α. It is unconventional because the learners are unfamiliar with equations such as this that combine trigonometric functions. (Teachers often focus on solving problems that their students will have to solve according to the curriculum, and such a problem was not part of the curriculum).

Using the circle applet, learners could identify that the equation describes a circle, and the circle varies according to the angle. In the present example, it is not difficult to understand the applet in the context of the problem. However, it was difficult for the ISMTs to solve the equation formally before they knew the solution.

This problem is presented unusually so that the learners are subjected to a high CL when trying to understand the essence of the problem, which includes multiple interactivity: think about circle properties, think about trigonometric functions, relate the radius of the circle to the size of the angle, think about cases where more than one solution is possible or where there is no solution at all, think about the problem through visual representations, think about the problem through algebraic representations, understand the interactivity and dynamic changes in the circle, and more. Based on the quantity of these elements, the problem could feasibly involve a high CL given that the external representation of the problem would be highly unfamiliar if not presented through the applet.

The assumption was that the many insights resulting from the interactive application increased the strategic flexibility of the learners, so that a wide variety of solutions emerged. Since the applet does not formally solve the problem—instead, it helps the user connect the many elements in the problem and reduce the number of interactive elements that create a dilemma—the dynamism presented by a changing circle increases the chance that the solver will investigate and understand the relationships between all the elements in the problem (circles and angles) and be able to connect the relationships to write a formal solution.

### 3.6. Data Sources

Data were qualitative and collected from four sources: (i) the ISMTs’ reflective journals, in which they documented their ideas, feelings, and insights during and directly after solving the problems—journal entries were semi-structured, prompted by a standing set of three questions printed in each participant’s journal booklet: (a) “What did you notice or discover while working with the applet?”, (b) “What was cognitively challenging, and why?”, and (c) “How might you use or adapt this problem or applet in your own teaching?”; participants were also free to add further reflections beyond these prompts; (ii) a self-scoring questionnaire with 21 statements (the ISMTs could add additional items if they so desired) that was completed after each problem (see detailed explanation below and the Appendix A); (iii) my own reflective journal, in which I recorded my thoughts when designing, teaching, and reflecting on the lessons; and (iv) audio recordings of the study sessions, which were transcribed verbatim (39 sessions in total). The use of multiple data sources allowed for triangulation of the findings, thereby supporting a coherent and trustworthy interpretation.

With respect to the questionnaire, this was inspired by CL measurement questionnaires from similar studies that were used to measure the three types of the CL and is based on the assumption that people can reliably recognize their cognitive processes and interpret their invested mental efforts (see Anmarkrud et al., 2019). Two expert researchers in mathematics education and cognitive load theory reviewed and validated the questionnaire. Note that the questionnaire was not used for statistical purposes; rather, it served to indicate to me, as both lecturer and researcher, which elements of the learning environment elicited CL for the learners in each lesson, thereby informing the design of subsequent lessons.

### 3.7. Data Analysis

#### 3.7.1. Questionnaires

The first two research questions, which concern the connection between the learning environment and teachers’ experience, were addressed through an analysis of the self-report questionnaires (Table A2) and diaries.

As noted, the questionnaires were not analyzed quantitatively. Instead, the focus was on the average scores assigned to each statement after each problem had been explored in the lesson. This aggregative, non-statistical approach to CL monitoring represents an underused but, I argue, pedagogically generative application of CL monitoring, and was explicitly designed to enable me to extract meaningful pedagogical information from the questionnaires—for instance, understanding why learners felt it necessary to add the statement “Lack of expertise in the use of applets” (Appendix A, statement 24) or why “Formally expressing the solution mathematically in writing” (statement 12) tended to generate “high CL” responses.

The analysis of the questionnaire data provided a clearer understanding of the events associated with each IWE-based problem, thereby informing the design of subsequent tasks to progressively reduce CL as the course advanced. At the end of the course, I organized all the statements (Table A2 in the Appendix A) and created summary graphs to identify which actions learners found most and least cognitively burdensome.

#### 3.7.2. Journals and Audio Transcripts

Although the study is primarily qualitative in its epistemological orientation, it incorporates a complementary layer of non-statistical quantitative data in the form of aggregated CL ratings from the self-report questionnaires. This mixed approach reflects the dual purpose of the instrument: to monitor the cognitive texture of each lesson and to provide descriptive evidence that contextualizes the qualitative findings. The qualitative findings were organized based on the research questions, with primary focus on the overarching question: How do in-service mathematics teachers experience and learn in a multimedia-supported professional development environment that uses interactive worked examples of non-routine mathematical problems?

After each lesson, the transcripts and the journal entries (my own and the ISMTs’) were divided into events. ATLAS.ti was then used to examine and note any statements related to the research questions. All statements were classified, sorted, and mapped as events based on four key themes linked to the research questions: (i) reducing CL by analyzing interactive WEs; (ii) analyzing interactive WEs as a problem-solving process; (iii) discovering new mathematical knowledge through interactive WEs; and (iv) uncovering new mathematical pedagogical/didactic knowledge or teaching practices, which were ultimately integrated into three main themes. The coding process was primarily inductive: initial codes emerged from close reading of the data without imposing a predetermined category scheme. Axial coding was then used to group the initial codes into the four themes listed above. Coding was conducted by the author–researcher. To establish trustworthiness, a second researcher experienced in mathematics education reviewed a randomly selected 20% subset of coded segments and independently applied the same codebook. Member checking was further employed: selected participants reviewed interpretive summaries of their journal excerpts and confirmed that the interpretations reflected their intentions.

## 4. Results

The qualitative analysis yielded three interconnected themes, each aligned with one of the overarching research questions. Table 2 provides a thematic overview mapping each theme to its theoretical grounding, primary data sources, and key findings. The sub-sections that follow present the evidence for each theme in detail.

### 4.1. Analyzing Interactive Worked Examples as a Problem-Solving Process

A significant initial challenge emerged at the outset of the course: many ISMTs perceived using interactive WEs as a first step in solving a problem to be “illegitimate” from a mathematical standpoint and that the traditional method (solving using pencil and paper) was the “proper” way to learn mathematics. It was, therefore, necessary to persuade the participants to follow the prescribed agenda. In other words, simply placing them in a setting that encourages learning and analysis was not enough to prompt them to analyze an interactive problem similar to the given one and then use that knowledge to solve the original problem. In fact, at the outset, this increased their CL, as evidenced by the data (from lessons and journals). This finding has direct implications for practice: professional development programs incorporating IWEs should devote explicit introductory time to legitimizing technology-mediated analysis as a form of mathematical inquiry, rather than assuming that participants will accept this mode of engagement from the outset.

It therefore became clear that intervention was necessary. This challenged me as both researcher and instructor: how could I persuade learners to confront and analyze a “ready-made” solution for a non-routine problem, to cultivate this novel habit of mind given the mental effort and CL involved in analyzing an interactive solution, and to apply the mathematical knowledge they gained to solving similar problems? However, once the ISMTs began analyzing the interactive solutions, they realized that the actions involved were similar to those needed to solve the original problem and eventually became convinced that using interactive WEs was a helpful tool for analyzing the problem and understanding the data and relationships involved.

The following statement, drawn from one ISMT’s reflective journal, illustrates this shift:

Initially, I was not convinced that analyzing an interactive solution was a substitute for solving the given mathematical problem [classically], but now…I understand the solution process. I found myself trying to identify the nature of the problem. After considering the strategy used [in the app] to solve the problem, I understood the sequence of the process. When the solution is presented interactively, the sequence [can be manipulated by] me. I then used this [experience] to write the solution in formal mathematical language according to my understanding, even though it may not have been precisely the thinking of the person who had designed the applet.

In other words, the ISMT’s initial reluctance to participate in an interactive learning environment was replaced by the realization that analyzing an interactive solution is, in itself, a problem-solving process (since it requires investing in analyzing the problem using the applet). Several other ISMTs expressed similar views, indicating that they were also beginning to see this environment as a legitimate mathematical activity and a way to learn how to solve problems directly. This aligns with one of Zaslavsky’s principles regarding dealing with conflicts, dilemmas, and problem situations: the very act of managing these situations transforms teachers into problem solvers in the broadest sense of the term.

Once this hurdle was overcome, the data suggested that the ISMTs tended to focus on five aspects when analyzing the interactive solution: (i) analyzing the problem; (ii) identifying the main idea and searching for sources of information and methods to solve the problem (theories, formulas, critical concepts, etc.); (iii) exploring considerations and strategies; (iv) selecting practical ideas; and (v) organizing ideas that appear effective for solving the problem most economically (reasons differed from one ISMT to another depending on their expertise and skills). They also considered eliminating ineffective ideas, such as those that prevent parsimonious solutions or might involve applying too many concepts.

Another example of the effectiveness of this method came during a discussion about a problem regarding the derivatives of tangents. An ISMT offered their insight that observing the process interactively with two other triangles led to their discovery that the perpendicular is always the derivative, which led to comprehending the reason behind this phenomenon, and which then led to the solution of the given problem.

Problems arose when the ISMTs sometimes did not understand the applets or the connection between the applet and the problem. Even more so, some expressed that figuring out what stood behind an application that someone else had built seemed more complicated. They also pointed out that having to connect all the information accumulated from the numerous static images seemed to require a high CL. However, the advantage of this method was that it increased familiarity with other possible solutions.

Indeed, individuals without appropriate expertise who are challenged to solve the problem may indeed experience high CL when the task is challenging and requires coping with complex problem-solving methods. However, the observations made by the ISMTs are consistent with studies claiming that a high internal CL is not necessarily detrimental if it is the result of heightened challenge and not heightened frustration (Sweller, 2015, 2016).

Although this shift in attitude (that using interactive WEs was a legitimate problem-solving method) occurred during the study itself, I became aware of it only retrospectively, after reading the journals and listening to the audio recordings. Analysis of the lessons suggested that the ISMTs avoided analyzing the applets until they realized that the analysis was an important part of the problem-solving process.

### 4.2. Discovering New Mathematical Knowledge Through Interactive Worked Examples

The findings suggest that interactive analysis enables ISMTs to acquire new mathematical knowledge by managing solutions and learning from numerous dynamic examples of the same problem. The technological tool allowed them to observe every step of the analysis, move to any part on which they chose to focus, examine relationships between different representations (graphical vs. algebraic, graphical vs. other graphical, etc.) and concepts (e.g., symmetry of integral areas, varied representation of trigonometry angles), identify connections between areas and graphical representations, and more. These relationships are usually difficult to visualize and practically impossible to represent simultaneously in the traditional manner. Analysis of the data from all sources revealed that the interactive solution (sometimes more than one) reduced CL and guided learners to carry out actions to understand the proof more deeply and then write it formally.

The following journal excerpt, written by one ISMT while engaging with the circle applet (see Section Example Problem and Applet), illustrates how the interactive analysis process facilitated the construction of new mathematical knowledge:

The given equation did not immediately look like any graph I was familiar with. However, the interactive applet displayed a variable circle, and this made me think that the equation might be describing a circle. Nevertheless, it was not clear to me how the trigonometric functions in the equation related to the equation of a circle. However, repeatedly playing with the circle applet made it clear to me that the trigonometric functions did have to do with calculating specific values. Nevertheless, my understanding was not complete. [Upon more experimentation], I carried out an in-depth analysis of some of the static images in the applet. This produced new knowledge about the concepts, both the procedures and the representations. For example, that the circle is a geometric environment that is determined by the values created from the relationship of the trigonometric functions representing the radius length. Therefore, the size of the circle varies, as does its position on the plane. The new procedures I discovered involve constructing the equation by connecting values derived from calculations using the sine and cosine, as well as translations of the circle on the plane. The new representations are the graphs of the sine function, which describe the circle, and the circle, which describes the trigonometric values. If it were not for the dynamic technological software, I would not have noted all these aspects. Later, when we solved the problem in the group, I noticed that whoever had tried to solve the equation and draw the required graph without using the applet either presented a partial solution or sketch for the equation or did not understand the connection between the trigonometric functions and the equation.

(see Figure 3)

This quotation and others show the ISMT’s process when building a schema for the solution. Even though they considered the dynamic tool something that gathered together the details, they appreciated that they were the ones who organized those details, connected them, and gave them new meaning. Thus, the dynamic tool functions as a form of “environmental storage,” freeing working memory resources—consistent with Sweller’s principle that well-designed external representations reduce internal cognitive load (Sweller, 1988). Also, as another ISMT said: “I don’t need to remember too much information at once. The tool ‘holds’ the information for me.”

It is also possible to generalize that the analysis of the app based on the experience of this ISMT conformed to Mayer’s “focused more is more” approach, that is, using design principles that motivate learners to cope with generative processing while at the same time focusing on reducing superfluous processing. The solution to the equation above is essentially simple: the content of the question was familiar, and the learners had all the knowledge necessary to solve the problem. Nevertheless, the problem is considered non-conventional because of its shape, style, and visual display. In other words, presenting it without the interactive applet would have led to high CL.

**Figure 4:**
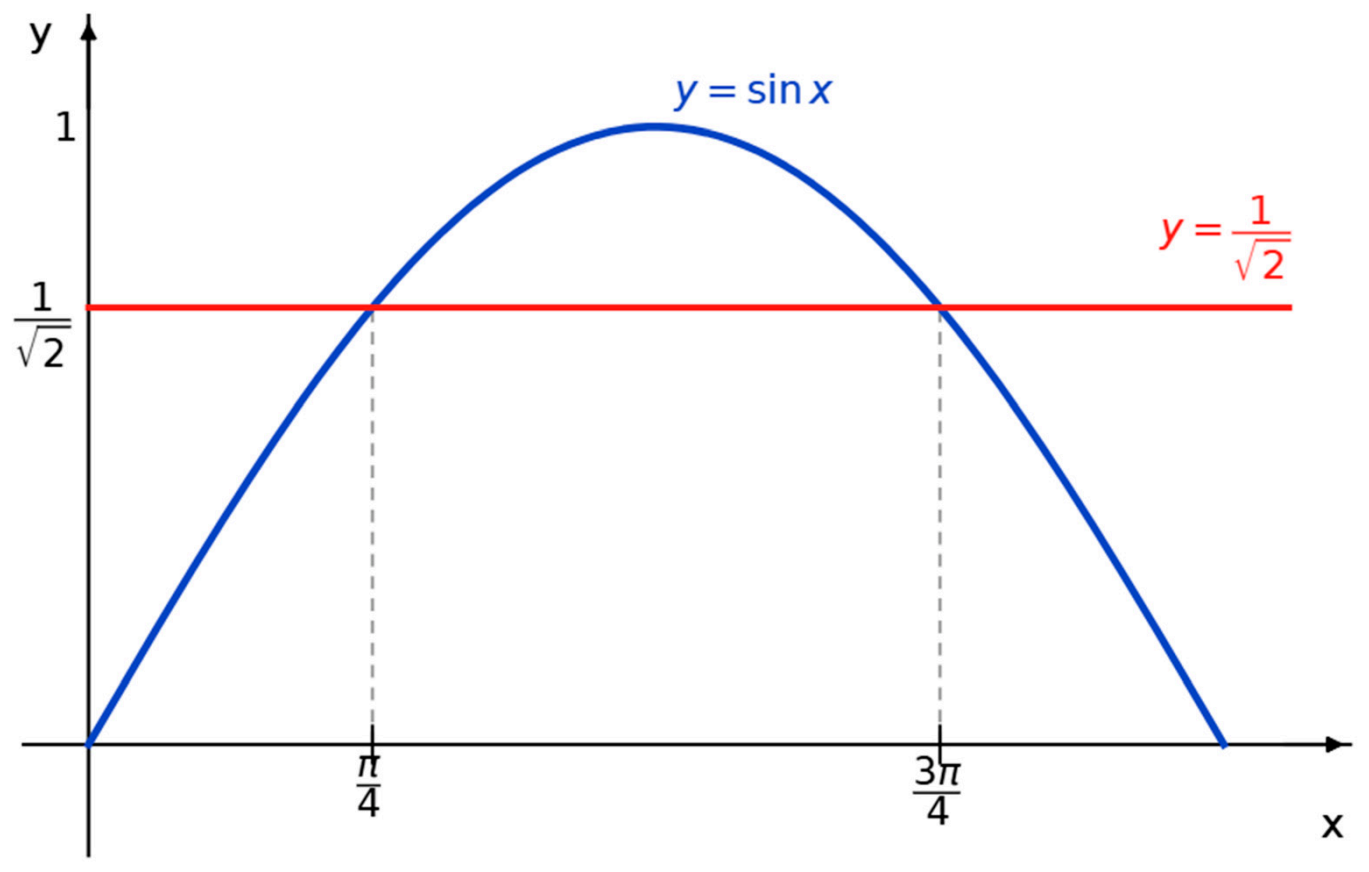
Graph showing the correct solution to the equation x^2^ + y^2^ + 4x·cos α + 8y·sin α + 10 = 0.

Even though the ISMTs had no opportunity to “play” with the interactive ruler, observing the applet still afforded them a new understanding of the relationship between the functions presented and their features, especially with respect to the relation(s) between the circle and the trigonometric functions. (For example, they could observe that the circle disappeared at different angle values, or that it existed in some quadrants on the plane but not in others (see Figure 2). It allowed them to follow the solution from stage to stage and from idea to idea, without arriving at a point where they might lose concentration, forget data, or miss other potential solutions. The applet presented extensive mathematical knowledge and freed them to acquire new knowledge even though it was never requested. In Figure 4, we can see that an ISMT investigated the graph of the sine function in a certain domain, regardless of the circle, to understand the function’s behavior in the domain.

When rating the CL of this problem, the ISMT wrote that they experienced a moderate-to-low load during their analysis of the solution, but a high load when writing the formal solution.

For this problem, the new mathematical knowledge obtained was the connection between a circle and trigonometric functions. The applet was not the “proof”; it was a tool by which to understand the mathematical phenomenon. None of the members of another group of students who did not participate in this study and did not have access to the interactive applet were able to sketch a figure that would lead to the solution of the problem and hence reach a complete solution, not only because this was an unfamiliar equation, but because, in essence, it is a dynamic event that is being expressed statically. (The “graph” combines a series of graphs of circles with standard features, each providing a partial solution to the problem).

Exploring the interactive example offered learners the chance to explain to themselves how the problem is solved (similar to previous studies by Mayer, 2014, 2021). This promoted their ability to understand the mathematical phenomena and concepts in greater depth, as noted by their reflections when they solved similar problems in the following lessons and attributed the knowledge and ideas they used specifically to this problem.

### 4.3. Discovering New Mathematical Pedagogical Knowledge for Teaching Practices

The ISMTs’ new mathematical knowledge led them to discuss appropriate ways of teaching “with themselves” (in their journals) and in the plenary. That is when they discovered that, as a result of the interactive example, they had learned about additional solutions that they had not previously been aware of, or when they discovered a novel way to understand a concept, they tried to convert this to didactic knowledge about appropriate teaching methods. For example, some of the ISMTs speculated how they might get their pupils to connect between the derivative of trigonometric functions and the unit circle (i.e., how they might use an applet based on a unit circle to illustrate problems related to the derivatives of tangents and cotangents), or whether they could use this problem to teach about other derivatives. Following are some excerpts from the class discussion that reflect the specific types of teachers’ knowledge described in Loewenberg Ball et al. (2008):

In the new curriculum and matriculation exams, there is a trend to present problems that require a better understanding of concepts, the link between representations, and proofs that commence by observing graphical representations.

(Knowledge of Content and Curriculum, Horizon Content Knowledge)

I wonder what is most appropriate for pupils. To first allow them to experiment with the interactive tool and then ask for the proof (as it was presented to me), or to first have them construct their proof in the traditional, formal way, and then introduce the applet and ask them to “prove” it with it?

(Knowledge of Content and Student [KCS])

Perhaps an idea would be to give them the applet alongside a static, properly written worked example to aid the transition between interactive worked examples and static worked examples and thus build new knowledge?

(Knowledge of Content and Teaching)

All the ideas seem good, didactically speaking, but the last seems to me to require the most CL because it is a transition between two worked examples and requires much concentration. However, the intense CL can lead them to deepen their understanding.

(KCS and Specialized Content Knowledge)

On the other hand, they are just [young] pupils, so perhaps it is better to let them play with the applet and follow that with a class discussion.

(KCS)

Such statements show the perceptions that the ISMTs developed regarding knowledge acquisition, CL theory, and teaching design. They understood the advantage of dealing with the type of problems they encountered in the course because they were allowed to experience how concepts, phenomena, and representations can be better understood in many contexts. They also understood that task design must consider their pupils’ prior knowledge and individual skills. The statements show that they understand that the better and more appropriately a task is designed, the better learners can extend their mathematical knowledge. Hence, some of them redesigned and posed problems inspired by the problems they had solved in the class and adapted them from a didactic perspective for their students.

From the ISMTs’ reflections, one can see that the task encouraged them to think about their teaching based on their students’ knowledge, in accordance with Zaslavsky’s principle regarding identifying and overcoming barriers to students’ learning, i.e., task construction must consider the students’ learning barriers and allow them to cope with these barriers. Learning barriers can include difficulty with various representations, a lack of prior knowledge, personal limitations, and more.

Here is an example of a problem one teacher designed:

- Look at the sine function that appears in the applet.
- Choose two significant features from the representation.
- Write down the features.
- Limit the function to a certain area-domain. Have the features changed?
- Draw a circle using the GeoGebra software. Try to move the circle’s center to different Cartesian plane quadrants.
- How did you change the circle equation to make this happen?
- Can you relate the sine function to the equation of a circle?

The ISMT who posed the problem reasoned that the design of the problem would gradually expose her students to the properties of each mathematical object and that only after studying each object would they be able to understand the relationship between a circle and a sine function. According to her, exposing them to the same applet she had encountered in class would not prompt them to conduct any experimentation and would only lead to frustration.

The data showed that the ISMTs were thinking about both themselves and their pupils at the same time. As they developed mathematically and gained valuable learning, they also focused on how to effectively share this knowledge and experience with their pupils, or how to appropriately adjust their experience for the pupils.

In the current study, using the scaffolding of interactive worked examples expanded and enriched the discussion beyond the learners’ personal experience.

### 4.4. Analysis of Questionnaire Results

The purpose of the questionnaires was to guide me in selecting appropriate classroom management practices. After each class, in which two mathematical problems were solved using different methods, ISMTs filled out a questionnaire. The total scores for the statements are listed in Appendix A. The number of responses to each statement varies because respondents occasionally omitted statements that were not relevant to their experience at the time. Accordingly, the totals reported in Table A2 aggregate ratings across all problem administrations, participants, and cohorts; because the number of responses differs across statements, these totals should be read as relative, descriptive indicators of where cognitive load concentrated rather than as counts intended for statistical inference. They rated each statement based on their experiences in solving the problems. If a specific activity was rated as increasing cognitive load, I assessed whether it was worthwhile to continue similar activities or replace them with ones that imposed a lower cognitive load. The underlying rationale was that if an activity increased cognitive load but was considered essential—such as “analyzing an unfamiliar problem using a partially solved example in an application”—it would be repeated. For example, the circle/trigonometry problem (Section Example Problem and Applet) was revisited across multiple sessions; by approximately the sixth lesson, ISMTs who had initially struggled with applet manipulation were independently adjusting the slider parameters and formulating written proofs without instructor prompting—indicating that CL had shifted from extraneous to germane. As ISMTs became more familiar with the activity, it became routine and the cognitive load decreased over time. Conversely, if an activity received a high cognitive load rating and was not essential for the course’s learning goals, I chose to omit it from subsequent lessons.

**Figure 5:**
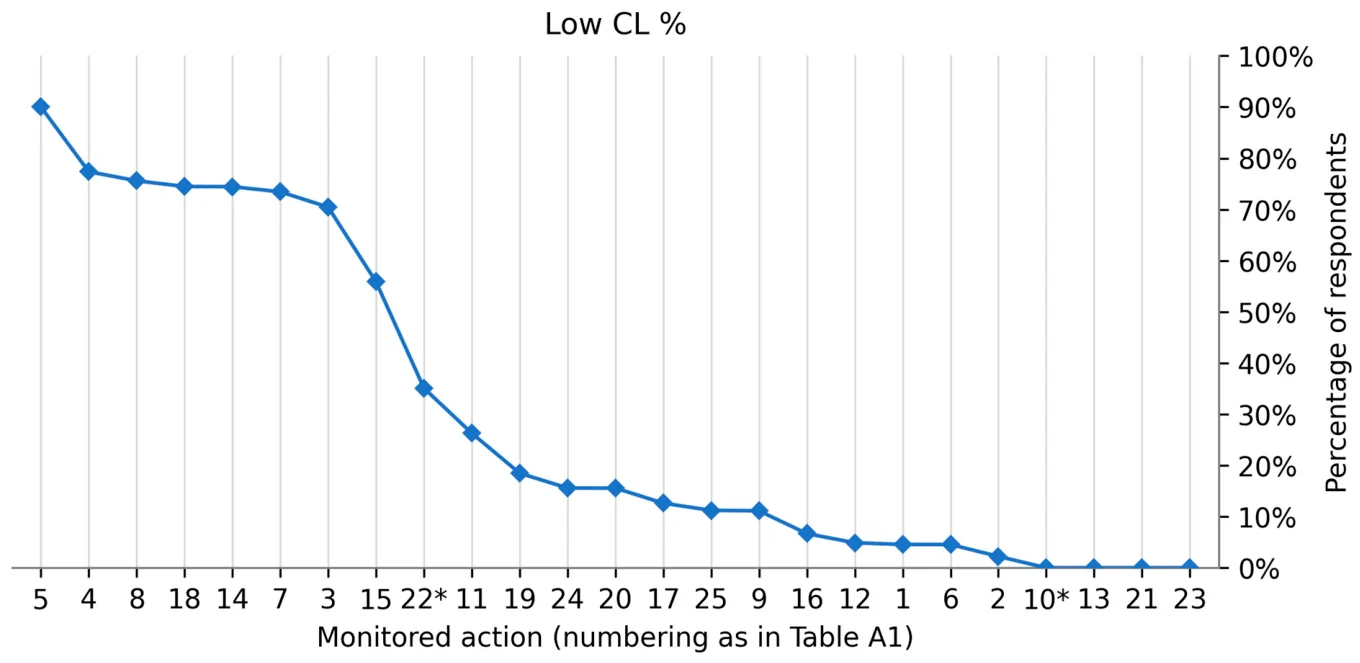
Low CL Rates. As can be seen, the actions that constituted a low cognitive load as the learning process progressed related to solving simple or familiar problems in familiar ways. Asterisks (*) mark activities whose cognitive-load rating changed sharply at some point during the course.

**Figure 6:**
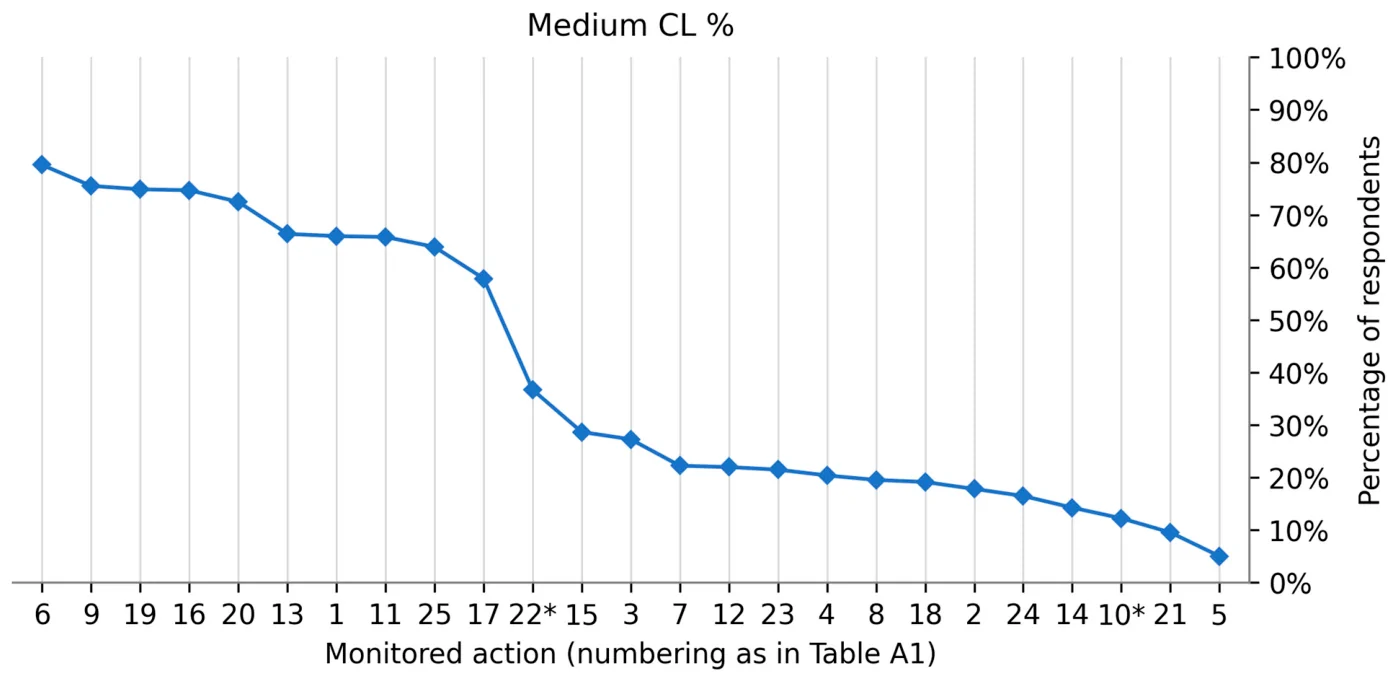
Medium CL Rates. Cognitive load increases when the actions are analyzed in interactive examples of less familiar problems. Asterisks (*) mark activities whose cognitive-load rating changed sharply at some point during the course.

**Figure 7:**
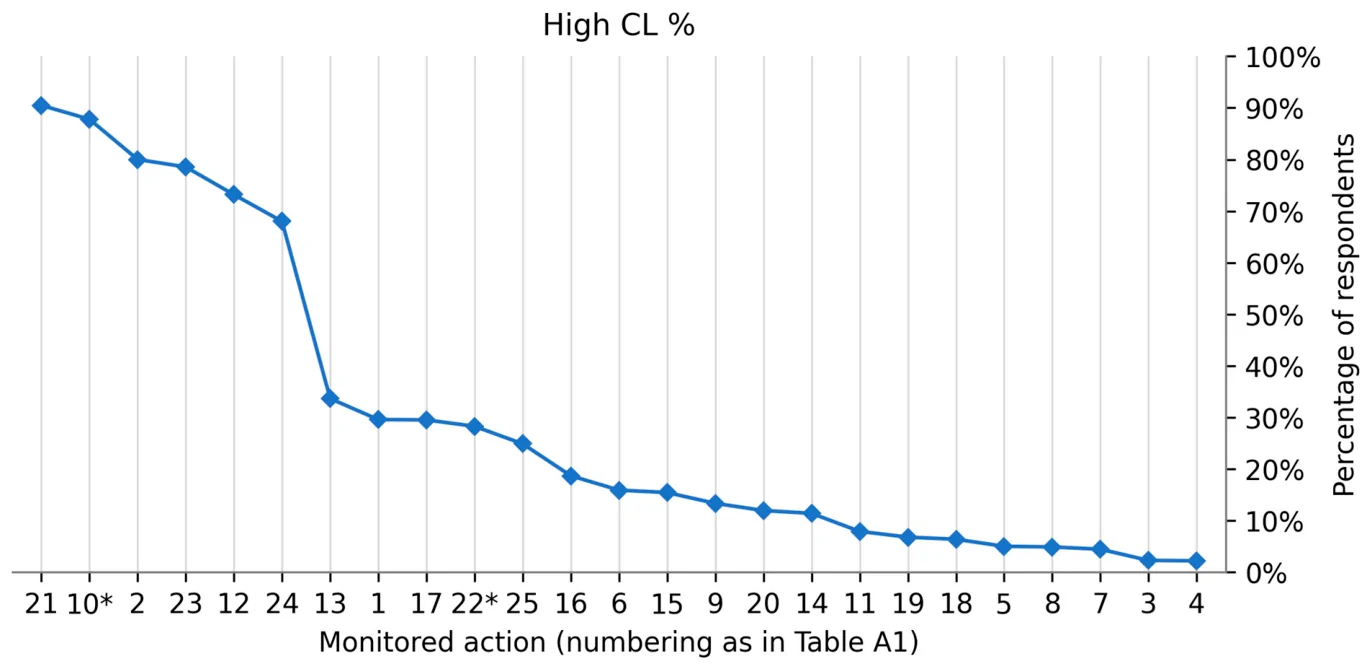
High CL Rates. As can be seen, the most CL is on the two actions: “Designing an interactive solution” and “Question expressed in an unfamiliar manner.” Asterisks (*) mark activities whose cognitive-load rating changed sharply at some point during the course.

Three graphs summarizing the course activity scores are presented below (Figure 5, Figure 6 and Figure 7). Each graph highlights the activities that received the highest CL ratings.

The aim was to engage teachers with unfamiliar problems and activities requiring skills they were still developing. Throughout the course, I focused on developing these two skills, incorporating these activities into nearly every lesson. Despite this frequent implementation, the activities still received a high rating. This persistent high-CL rating for “Designing an interactive solution” is particularly noteworthy. Unlike most activities whose CL declined with familiarity, this task remained cognitively demanding across all three cohorts. This suggests that designing an IWE is not a skill that becomes routine through mere repeated exposure; it may represent a genuinely high-interactivity task requiring sustained integration of mathematical content knowledge, technological expertise, and pedagogical reasoning simultaneously. From a CLT perspective, this task may impose irreducible intrinsic load because its core demands are inseparable. This finding implies that if practitioners wish to equip teachers to design their own IWEs—a desirable long-term outcome—they should provide dedicated scaffolding for the design process itself (cf. Ovadiya, 2021; Ovadiya & Kontorovich, 2026), distinct from the scaffolding provided for engaging with existing IWEs.

To clarify the pattern already visible in the per-level summaries (Figure 5, Figure 6 and Figure 7; Table A2), the 25 monitored actions can be grouped by the type of cognitive activity they involve. This grouping reveals a consistent gradient (summarized in Table A3). Actions that involved analyzing existing interactive worked examples were rated lowest in cognitive load (mean CL Index 1.60), whereas actions involving unfamiliarity or gaps in expertise were rated highest (mean 2.74); solving and expressing solutions without an interactive example, and designing new material, fell in between (means 2.26 and 2.21, respectively). Importantly, the “designing” grouping was not uniform: designing an interactive solution (item 10) was among the most demanding actions in the entire set, whereas designing on paper or drafting follow-up questions was only moderately demanding. This pattern is consistent with the interpretation that it is the integration of mathematical, technological, and pedagogical demands specific to interactive-example design—rather than design in general—that imposes the highest, least-reducible load. These proportions are descriptive summaries of the monitoring questionnaire and are not intended to support statistical inference.

**Figure 8:**
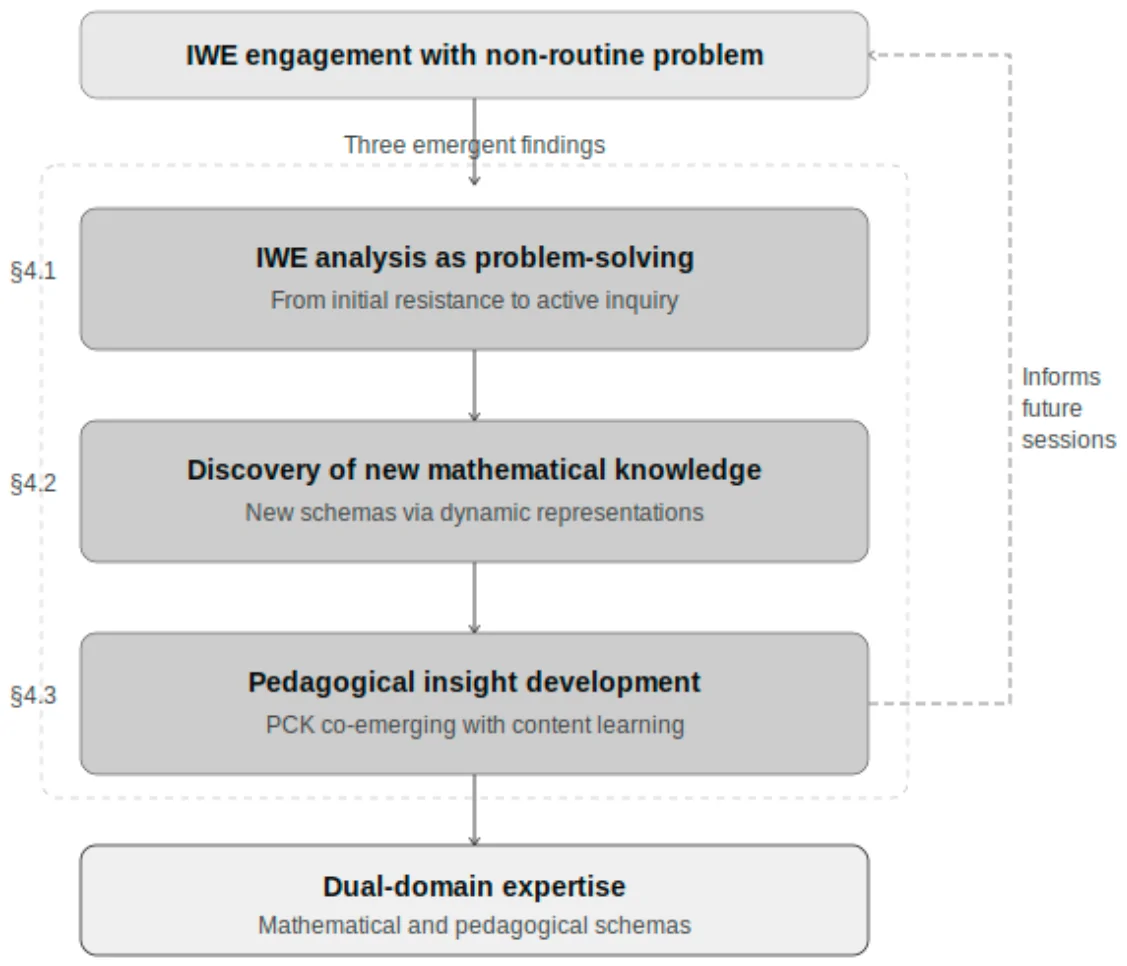
The emergent learning process: developmental flow from IWE engagement through three interconnected findings to dual-domain expertise (i.e., the simultaneous development of mathematical content knowledge and pedagogical content knowledge), with a feedback loop informing future sessions.

Taken together, these three thematic findings describe an emergent developmental process, which is summarized in Figure 8.

## 5. Discussion

The literature refers to teachers’ experiences and their reflectivity as a critical development process (Brookfield, 2017; Schön, 2017). Brookfield (2017) notes that reflective elements influence and empower teaching, including events in which teachers discover new knowledge and design their forthcoming lessons based on their experiences, insights, and conclusions. A similar phenomenon occurred in the current study: the documentation and interpretation of the learning experience empowered the teachers by providing important insights about learning and teaching.

The first insight from the results was that analyzing an interactive solution could be a problem in itself. This was demonstrated when the ISMTs were first asked to investigate and analyze the applet in the context of solving the problem. However, observing how the ISMTs reconsidered their perception of problem-solving and accepted that analyzing an applet could be a legitimate stage was an interesting finding, as ISMTs need to be flexible in finding ways to present problem-solving situations. It is interesting since some ISMTs had experienced learning that did not necessarily link the concepts of actions and representations. Thus, their current experience prompted them to create a mathematical understanding of terms, actions, and representations. According to studies, teachers may design events to analyze problems that are similar to those they encountered in their learning (Boaler, 2015; Gadanidis et al., 2016; Kontorovich & Ovadiya, 2023).

What about CL? When the ISMTs still resisted using interactive WEs, they labeled the activity “high CL.” However, once they had accepted that working with applets was a legitimate activity, they labeled the CL as appropriate for the problem’s mathematical complexity level of the specific problem interactive elements. That is, the initial emotional resistance to the tool—rooted in skepticism about its legitimacy as a valid problem-solving medium—suppressed the cognitive load reduction that the technology might otherwise have provided. Perhaps ISMTs can be encouraged to develop mental flexibility by teaching them using repeated non-routine teaching methods that they find meaningful.

This emotional trajectory—from initial resistance and frustration toward engagement and eventual sense of accomplishment—aligns with the theoretical framework proposed by Weber et al. (2023), who found that positive emotions predict germane cognitive load and immersion, while negative emotions suppress both. In the present study, the ISMTs’ initial labeling of applet interaction as “illegitimate” reflected an affective barrier that temporarily prevented them from directing cognitive resources toward learning. Once this barrier was overcome, their emotional experience shifted, and their self-reported cognitive load came to reflect the actual mathematical complexity of the task rather than emotional resistance to the tool. This pattern suggests that in IWE-based professional development, the management of teachers’ affective responses to unfamiliar digital tools (Ovadiya, 2023, 2025) is not peripheral to the cognitive design of instruction but is an integral component of it. Ensuring that teachers experience early moments of success with the applet—through structured orientation or graduated task complexity—may reduce the affective interference that initially suppresses productive engagement.

The second insight relates to the ability of interactive examples to aid the learner in generalizing the concept under study and any subsequent questions raised as a result of constructing a generalized schema. According to Castro-Alonso et al. (2015) and Mayer (2021), dynamic representations can motivate learning, but there is a risk that the information they present may be superficial. They recommend manipulating elements while learning through dynamic visual representations to overcome this. Indeed, in the current study, the ISMTs had to manipulate elements in the interactive solution because the solution was not presented to them as “a finished video” but as a tool whose dynamics they could control and manage. They could manipulate the interactive example in the applet and track its components, meaning that they had the power to select which components to focus on and analyze and which to ignore.

In addition, not every interactive example presented the complete solution; sometimes, it merely illustrated a key idea needed to solve the problem. This situation encouraged the ISMTs to investigate the applet and decide what was critical for the solution, leading to a deeper understanding of the mathematical aspects of the problem and valuable discussions about creating similar, appropriate environments for teaching how to solve mathematical problems.

“Experts have acquired schemas which play a crucial role in how they approach and solve problems” (Sweller, 1988, p. 259). “Since schema acquisition is possibly the most important component of problem-solving expertise, the development of expertise may be retarded by a heavy emphasis on problem-solving”.

(Sweller, 1988, p. 284)

In some of the events, the teachers indicated a high CL score for elements that contributed to the experience of solving the problem. Their journals, together with my own, documented rich learning experiences that included lively discussions reflecting their new mathematical and pedagogical knowledge. This suggests that a high CL can sometimes indicate an intellectual effort that presents a positive challenge, allowing them to overcome it and contribute to their knowledge.

This finding resonates with the recent theoretical synthesis by Zou et al. (2025), who argue that CLT’s traditional assumption of a linear progression from cognitive overload to learning failure is inadequate for capturing the complexity of real-world learning environments. They propose that brief periods of elevated cognitive load—particularly when supported by well-designed interactive tools that enable cognitive offloading—can facilitate deeper schema construction rather than impeding it. In the present study, the applets served precisely this function: by externalizing dynamic mathematical representations, they redistributed the cognitive demands of the task, enabling teachers to sustain engagement with genuinely complex content without experiencing unproductive overload. The interactive nature of the applets transformed what might otherwise have been an overwhelming cognitive burden into a manageable, exploratory experience. This perspective suggests that the goal of IWE design in teacher professional development should not be to eliminate cognitive challenge but to calibrate it—ensuring that the load experienced is predominantly intrinsic and germane, directed toward mathematical understanding and pedagogical insight, rather than extraneous and arising from poorly managed instructional design.

The learning environment also allowed the ISMTs to learn through experimentation, interaction, and the use of external sources and to reflect on their own teaching practices consciously. They learned that analyzing the applet requires considering both static and dynamic mathematical conditions to understand the most important ideas presented. Such analysis can be equivalent to solving mathematical problems in the classical sense. The environment enabled the ISMTs to acquire new mathematical knowledge through class dialogue and their written reflections.

The sense of success the ISMTs experienced while analyzing the applets altered their beliefs about what a problem-solving environment can be, and they realized that analyzing an interactive applet is a problem-solving process in its own right. This may alter the way they practice teaching, as a shift in teachers’ ideas about an activity or teaching method often leads to a corresponding change in their teaching practice (Lane & Ní Ríordáin, 2020).

Their responses to the research environment indicate a plan to modify the teaching environments in their classrooms. This echoes the results of a study in physics classes regarding how a dynamic multimedia environment affected the understanding of lenses (Dervić et al., 2019). In this case, the interactivity between the learner and the applet reduced the CL of the learners, thereby allowing them to understand the physical phenomenon in greater depth.

Finally, Sweller et al. (2019), summarizing twenty years of research on CL, pointed out that students at all levels can be taught to apply CL theory principles to manage their CL. This is what happened in the current study. The repeated action of filling out the questionnaire after each solution, along with discussions amongst ISMTs themselves and in groups about the CL they had indicated for each statement, led the ISMTs to reflect and understand what elements increased or decreased CL. It fostered their ability to manage these elements. The ISMTs’ understanding of their processes led them to discuss what processes their students underwent.

In conclusion, this study applied cognitive psychology research to the pedagogy of a teacher professional development program. It showed that teachers benefit from learning environments designed according to the structure of human cognition (Sweller), multimedia use guidelines in teaching (Mayer), and properly designed task management strategies (Zaslavsky). A note of interpretive caution is warranted, however, regarding the attribution of pedagogical insight development specifically to the IWEs. The course also incorporated plenary discussions, collaborative reflection, and repeated self-assessment through the CL questionnaire; any of these elements, independently or in combination, may have contributed to the emergence of pedagogical thinking. While the journal data do contain instances in which ISMTs explicitly connect a pedagogical idea to their immediate experience with the applet (see Section 4.3), the study design does not allow a definitive causal attribution. Future research employing comparison conditions or more fine-grained trace data would be needed to isolate the contribution of the IWE format per se from the broader learning environment.

## 6. Conclusions

This study examined how 30 in-service mathematics teachers engaged with interactive worked examples of non-routine problems in a professional development setting grounded in cognitive load theory, multimedia learning theory, and task-design principles. The findings reveal three key insights. First, teachers came to perceive the analysis of interactive solutions as a legitimate and active form of problem-solving, not merely passive observation. Second, the dynamic, interactive format enabled teachers to discover new mathematical relationships and develop deeper conceptual understanding that would have been inaccessible through traditional static presentations. Third, teachers simultaneously and naturally developed pedagogical content knowledge—reflecting on how to adapt and present these experiences for their own students.

These findings suggest that carefully designed interactive worked examples can serve as cognitively efficient tools for dual-domain professional learning, supporting both mathematical and pedagogical development concurrently. More broadly, the study advances a claim worth stating plainly: the analysis of a well-designed interactive example is not a diluted substitute for problem-solving but a form of problem-solving in its own right—and one capable of building mathematical and pedagogical schemas at the same time. I offer this as a conceptual contribution grounded in rich qualitative evidence, while readily acknowledging that its causal and generalizable status awaits the comparative and longitudinal tests outlined below. The CL questionnaire data further indicate that activities perceived as high-load but intellectually rewarding (such as designing interactive solutions or tackling unfamiliar problem formats) remained persistently challenging throughout the course, pointing to their enduring value as developmental targets. Future research should examine the long-term effects of such professional development on classroom practice and explore how these findings transfer across different mathematical domains and educational contexts. Several limitations of this study warrant acknowledgment. First, the sample comprised 30 in-service mathematics teachers drawn from a single graduate course at one institution, which limits the generalizability of the findings. Second, as practitioner–researcher, I occupied the dual roles of instructor and researcher throughout the study (see Ovadiya, 2024; Ovadiya & Goldberg, 2025). While ongoing self-documentation and the use of multiple data sources were employed to address potential reflexivity, this dual positioning may nonetheless have introduced some degree of observer effect. Because the participants were my own students, their self-reports may also have been shaped by social-desirability and power dynamics inherent in the teacher–student relationship; accordingly, I interpret the findings as a situated, illustrative account rather than as generalizable claims. Third, the reporting of findings centers on a single illustrative problem, which, while enabling detailed analysis, does not fully represent the breadth of the 26-problem curriculum. With respect to implications for practice, the results suggest that professional development programs designed around the principles of cognitive architecture (Sweller), multimedia learning (Mayer), and purposeful task design (Zaslavsky) can provide a cognitively efficient and pedagogically rich environment for teacher learning. Specifically, incorporating IWEs into professional development curricula appears to enable teachers to advance their subject matter expertise and develop pedagogical insights concurrently—a dual benefit that is difficult to achieve within a single instructional format. The CL self-assessment instrument introduced in this study may also be of practical value to other educators as a low-stakes, formative tool for monitoring and adapting instruction in real time. Future research directions include the following: (a) longitudinal investigations of whether and how participation in IWE-based professional development translates into sustained changes in teachers’ classroom practice; (b) exploration of the generalizability of these findings across other STEM disciplines and across diverse teacher populations; (c) experimental or quasi-experimental studies that compare the effectiveness of IWE-based professional development with more conventional approaches; and (d) investigation of how professional learning communities might serve as ongoing support structures to sustain and deepen the gains initiated in IWE-based courses.

## Figures and Tables

**Figure 3:**
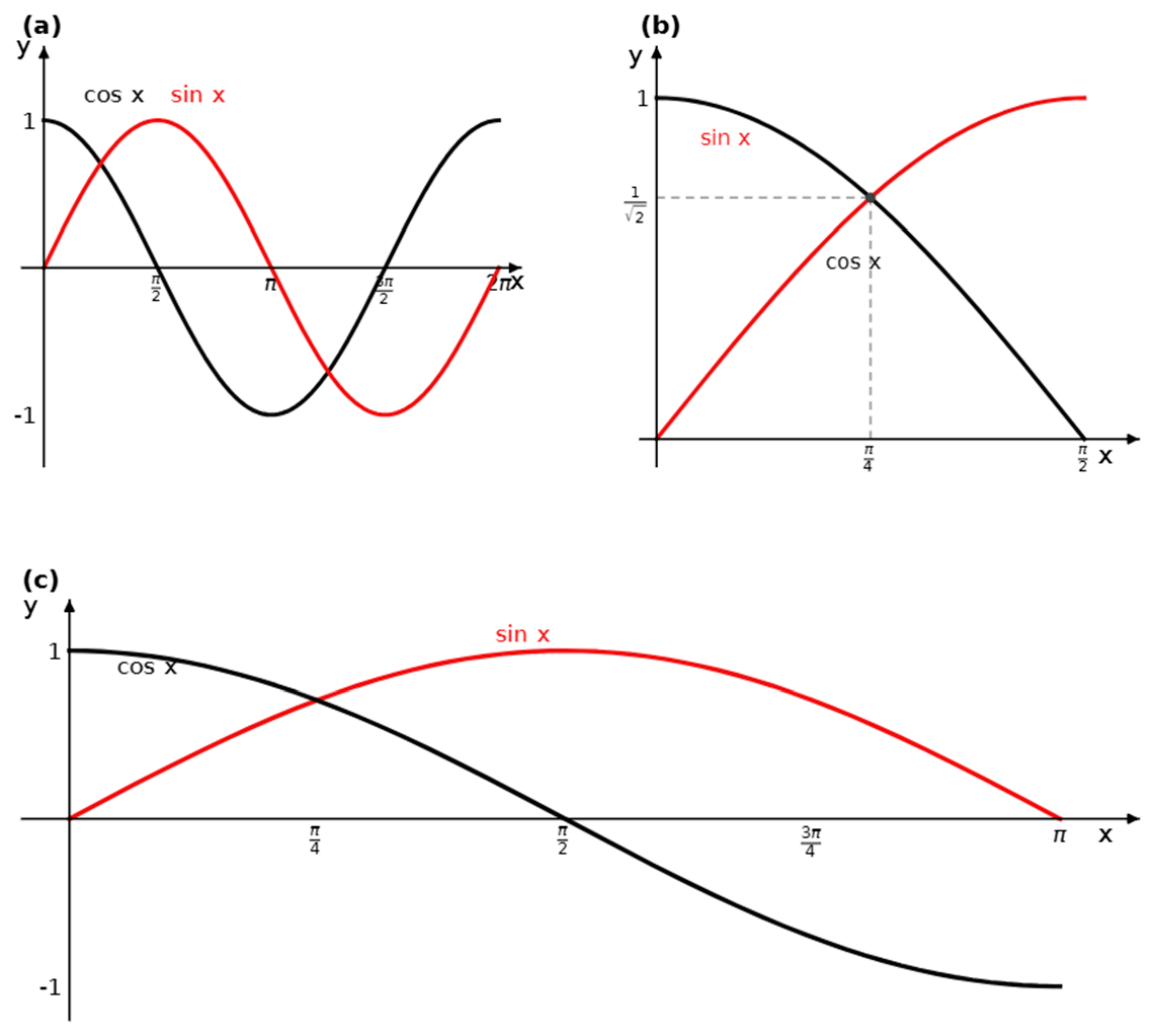
Sketch of a graph made when the applet was not observed.

**Table 1:** The three research questions.

Theoretical Framework	Core Theoretical Constructs	Aligned Research Question
Cognitive Load Theory (Sweller, 2015)	Human cognitive architecture; intrinsic, extraneous, and germane cognitive load; instructional efficiency	How do interactive worked examples of non-routine mathematical problems influence the cognitive processing of in-service mathematics teachers during their learning?
Multimedia Learning Theory (Mayer, 2014, 2021)	Selection, organization, and integration of visual and verbal information; dual-channel processing; generative learning	How do multimedia-based interactive tasks support the selection, organization, and integration of mathematical knowledge by in-service teachers?
Task Design for Teacher Learning (Zaslavsky, 2008)	Task adaptability; identifying similarities and differences; coping with pedagogical dilemmas; promoting reflection and flexible thinking	What features of the interactive learning environment promote teachers’ adaptability, awareness of mathematical structures, and ability to cope with pedagogical dilemmas?
Overarching research question	How do in-service mathematics teachers experience and learn within a multimedia-supported professional development environment that uses interactive worked examples of non-routine mathematical problems?	

**Table 2:** Thematic overview of results: alignment between theoretical grounding, data sources, and key findings across the three themes.

Theme	Theoretical Grounding	Primary Data Sources	Key Finding
IWE analysis as problem-solving (Section 4.1)	CLT; Zaslavsky (2008)—coping with dilemmas	Reflective journals; audio transcripts; CL questionnaires	Initial resistance (“illegitimate” tool) gave way to recognition that applet analysis is itself an active problem-solving process, requiring strategy selection, hypothesis testing, and schema construction.
Discovery of new mathematical knowledge (Section 4.2)	CLT—cognitive offloading; Mayer (2021)—multimedia design	Reflective journals; sketches and written solutions; CL questionnaires	Dynamic representations enabled ISMTs to construct relationships between mathematical objects (e.g., circles and trigonometric functions) that were inaccessible through static or pencil-and-paper approaches; moderate-to-low CL during analysis, higher CL when formalising the solution.
Development of pedagogical insight (Section 4.3)	Zaslavsky (2008)—task adaptability; Loewenberg Ball et al. (2008)—MKT	Reflective journals; plenary discussion transcripts; teacher-designed problems	Mathematical learning concurrently generated pedagogical reasoning (KCS, KCT, HCK); ISMTs spontaneously designed adapted tasks for their own students, grounded in the insights gained through IWE engagement.

**Figure A1:**
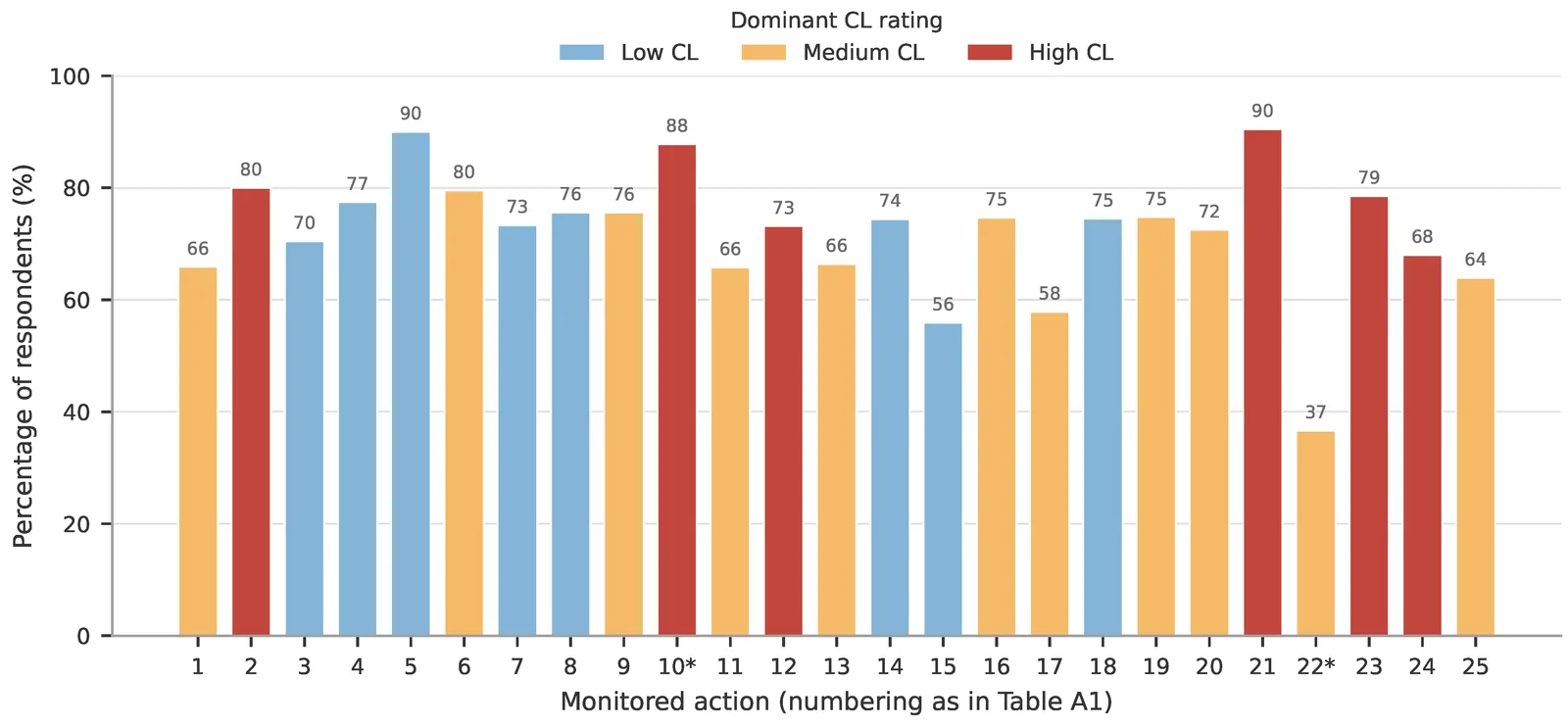
Average overall cognitive load ratings for all 25 questionnaire statements, aggregated across all participants and lessons throughout the course. Asterisks (*) mark activities whose cognitive-load rating changed sharply at some point during the course.

**Figure A2:**
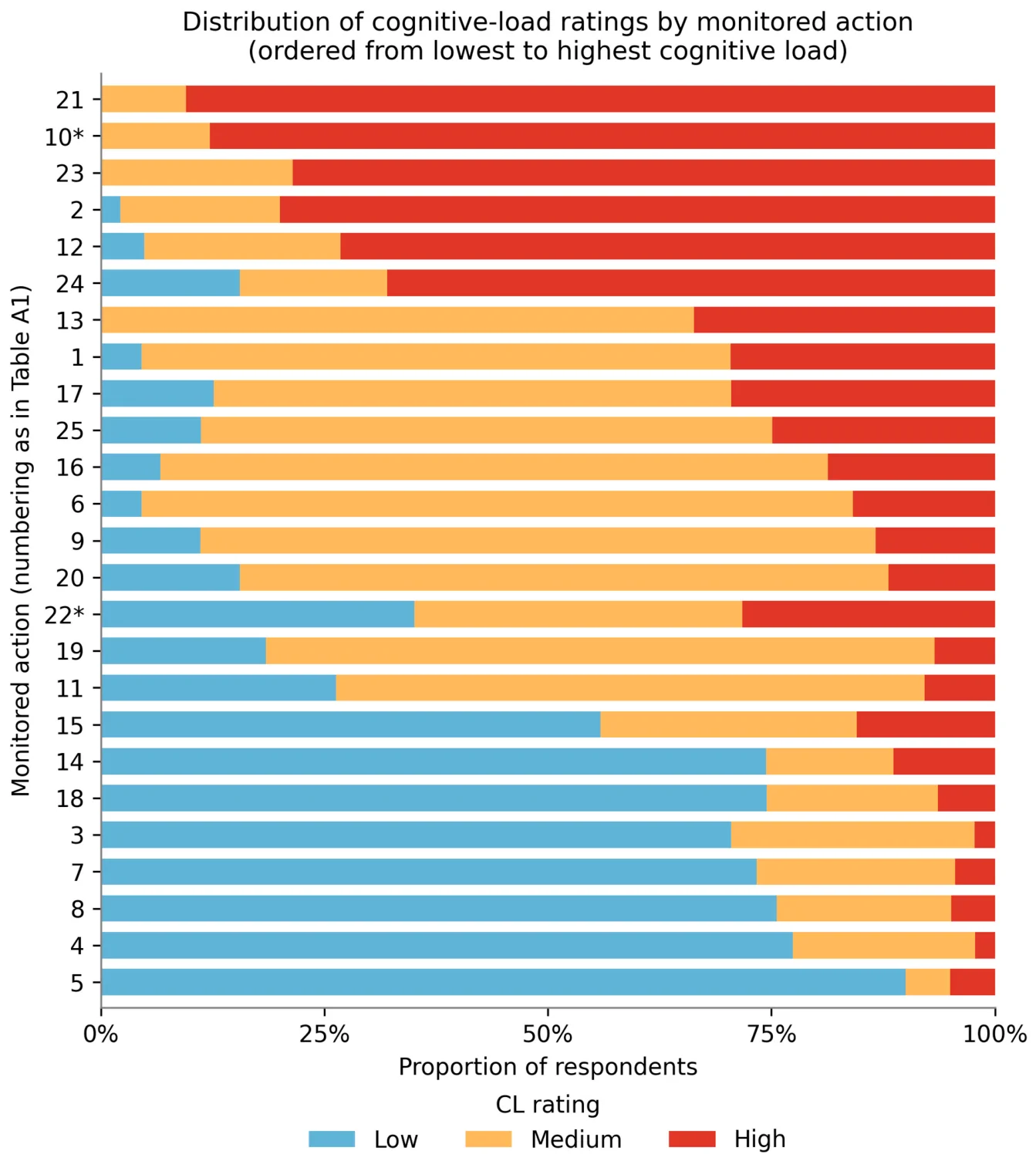
Distribution of cognitive-load ratings by monitored action (100% stacked), ordered from lowest to highest cognitive load. This consolidated, high-resolution view corresponds to the per-level summaries shown separately in Figure 5, Figure 6 and Figure 7. Asterisks (*) mark activities whose cognitive-load rating changed sharply at some point during the course.

## Data Availability

The questionnaire summary data is in the appendices. Other data is not available to maintain participant privacy.

## Abbreviations

The following abbreviations are used in this manuscript:

CL	Cognitive Load
CLT	Cognitive Load Theory
IWE	Interactive Worked Example
ISMT	In-Service Mathematics Teacher
PCK	Pedagogical Content Knowledge
WE	Worked Example

## Appendix A

**Table A1:** CL Self-Rating Instrument Administered to ISMTs After Each Problem. This table presents the rating instrument as distributed to participants; it was completed individually after each problem and served as a tool for course monitoring (see Table A2 for the aggregated totals). The columns Low CL, Med CL, and High CL were left blank by design for participants to fill in.

#	Actions and Learning Processes	Low CL 1–3	Med CL 4–5	High CL 6–9
1	Solving a familiar problem without a worked example			
2	Solving a problem when the solution is shown in an unfamiliar style			
3	Deciphering a non-interactive example for a familiar problem			
4	Deciphering a non-interactive example for a non-familiar problem			
5	Deciphering an interactive worked example for a familiar problem			
6	Deciphering an interactive worked example for a non-familiar problem			
7	Deciphering a specific part of an interactive worked example			
8	Deciphering an interactive worked example for a close similar problem			
9	Deciphering an interactive worked example for a far similar problem			
10	Designing an interactive solution *			
11	Designing a solution in writing (pencil and paper)			
12	Formally expressing the solution mathematically in writing			
13	Explaining the solution using interactive worked example and technological tools (applets)			
14	Using interactive worked example to manage the applet			
15	Using interactive worked example and self-regulation			
16	Emotions, stress, and uncertainty in problem solving			
17	Emotions, stress, and uncertainty in investigating non-familiar interactive worked example problem solving			
18	Emotions, stress, and uncertainty in investigating familiar interactive worked example problem solving			
19	Time pressure			
20	Environmental pressure (e.g., others have completed the task and I haven’t)			
21	Question expressed in an unfamiliar manner			
22	Designing additional questions that may be connected to the interactive solution *			
23	Lack of expertise in the area of the problem			
24	Lack of expertise in the use of applets			
25	(Additional criteria added by students)			

# denotes the serial number of the action or learning process. * The two scores indicate that the scores changed sharply at some point during the course.

**Table A2:** Aggregated CL Scores for All Statements Across All Anonymous Questionnaires. The final column reports the activity category assigned to each statement.

#	Actions and Learning Processes	Low CL 1–3	Med CL 4–5	High CL 6–9	Category
1	Solving a familiar problem without a worked example	26	377	169	Solving/expressing
2	Solving a problem when the solution is shown in an unfamiliar style	13	104	468	Solving/expressing
3	Deciphering a non-interactive example for a familiar problem	403	156	13	Analyzing existing
4	Deciphering a non-interactive example for a non-familiar problem	494	130	14	Analyzing existing
5	Deciphering an interactive worked example for a familiar problem	468	26	26	Analyzing existing
6	Deciphering an interactive worked example for a non-familiar problem	26	455	91	Analyzing existing
7	Deciphering a specific part of an interactive worked example	429	130	26	Analyzing existing
8	Deciphering an interactive worked example for a close similar problem	403	104	26	Analyzing existing
9	Deciphering an interactive worked example for a far similar problem	65	442	78	Analyzing existing
10	Designing an interactive solution *	0	65	468	Designing new
11	Designing a solution in writing (pencil and paper)	130	325	39	Designing new
12	Formally expressing the solution mathematically in writing	26	117	390	Solving/expressing
13	Explaining the solution using IWE and technological tools (applets)	0	310	157	Analyzing existing
14	Using IWE to manage the applet	340	65	52	Analyzing existing
15	Using IWE and self-regulation	250	128	69	Analyzing existing
16	Emotions, stress, and uncertainty in problem solving	35	392	98	Solving/expressing
17	Emotions, stress, and uncertainty in investigating non-familiar IWE problem solving	65	298	152	Analyzing existing
18	Emotions, stress, and uncertainty in investigating familiar IWE problem solving	339	87	29	Analyzing existing
19	Time pressure	98	397	36	Solving/expressing
20	Environmental pressure (e.g., others have completed the task and I haven’t)	82	382	63	Solving/expressing
21	Question expressed in an unfamiliar manner	0	10	95	Unfamiliarity/expertise
22	Designing additional questions connected to the interactive solution *	180	188	145	Designing new
23	Lack of expertise in the area of the problem	0	35	128	Unfamiliarity/expertise
24	Lack of expertise in the use of applets	87	92	380	Unfamiliarity/expertise
25	Motivation and ideas to solve the problem in a non-digital way	64	365	142	Solving/expressing

# denotes the serial number of the action or learning process. * The two scores indicate that the scores changed sharply at some point during the course.

**Table A3:** Cognitive-load profile by activity category (a descriptive summary of the categories assigned in the final column of Table A2). For each category, the table reports the number of monitored actions and the mean proportion of Low, Medium, and High cognitive-load ratings, with a mean CL Index (Low = 1, Medium = 2, High = 3; range 1–3). Values are descriptive summaries of the monitoring questionnaire and are not intended to support statistical inference.

Activity Category	No. of Actions	Mean Low %	Mean Med %	Mean High %	Mean CL Index
Analyzing existing	12	52%	36%	12%	1.60
Designing new	3	20%	38%	41%	2.21
Solving/expressing	7	9%	56%	35%	2.26
Unfamiliarity/expertise	3	5%	16%	79%	2.74
